# Assessing of growth, antioxidant enzymes, and phytohormone regulation in *Cucurbita pepo* under cadmium stress

**DOI:** 10.1002/fsn3.2169

**Published:** 2021-01-31

**Authors:** Oumayma Labidi, Vicente Vives‐Peris, Aurelio Gómez‐Cadenas, Rosa M. Pérez‐Clemente, Noomene Sleimi

**Affiliations:** ^1^ RME‐Laboratory of Resources, Materials and Ecosystems Faculty of Sciences of Bizerte University of Carthage Bizerte Tunisia; ^2^ Departmento de Ciencias Agrarias i del Medi Natural Universitat Jaume I Castello ´de la Plana Spain

**Keywords:** antioxidant enzymes, Cd‐tolerance, *Cucurbita pepo*, hormonal regulation, malondialdehyde, proline

## Abstract

One of the major problems worldwide is soil pollution by trace metal elements, which limits plant productivity and threatens human health. In this work, we have studied the effect of different concentrations of cadmium on *Cucurbita pepo* plants, evaluating different physiological and biochemical parameters: hormone signaling, metabolite concentration (malondialdehyde and hydrogen peroxide) and, in addition, the antioxidant enzyme activities of catalase and superoxide dismutase were evaluated. The production of biomass decreased under the Cd‐stress. The results showed that *C. pepo* accumulates higher amounts of Cd^2+^ in roots than in shoots and fruits. Cd^2+^ differently affected the content of endogenous phytohormones. Furthermore, data suggest an essential involvement of roots in the regulation of tolerance to trace elements. As a result, indole acetic acid content increased in roots of treated plants, indicating that this phytohormone can stimulate root promotion and growth under Cd‐stress. Similarly, salicylic acid content in roots and shoots increased in response to Cd^2+^, as well as abscisic acid levels in roots and fruits. In roots, the rambling accumulation pattern observed for jasmonic acid and salicylic acid suggests the lack of a specific regulation role against trace element toxicity. The activity of catalase and superoxide dismutase decreased, disrupted by the metal stress. However, the proline, malondialdehyde and hydrogen peroxide content significantly increased in Cd^2+^in all the analyzed tissues of the stressed plants. All these data suggest that *C. pepo* plants are equipped with an effective antioxidant mechanism against oxidative stress induced by cadmium up to a concentration of 500 μM.

## Graphical abstract

*Cucurbita pepo* has a strong ability to tolerate cadmium by its preferentially accumulation in the roots. Trace metal elements tolerance was determined by the barrier function of root system in *C. pepo*. Indole acetic acid may be involved in the tolerance of *C. pepo* to trace element stress by stimulating root promotion and growth.

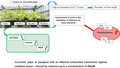

## 1 INTRODUCTION

A major environmental problem, generated by industrial emissions and urban wastes due to human activities, is the soil, water, and air contamination by a variety of organic and inorganic components (Ozaki et al., 2019).

Some metallic elements, such as iron (Fe), manganese (Mn), zinc (Zn), copper (Cu), molybdenum (Mo), and nickel (Ni), are essential for the organism functionality, but their increased concentration can be dangerous for fauna and flora. Other nonessential elements for living organisms, as lead (Pb), mercury (Hg), and cadmium (Cd), are also considered toxic even at low concentrations. (Awa & Hadibarata, 2020). Their presence in soil is greatly affected by industrial and anthropogenic activities (Alkorta et al., 2004).

It has been reported that some plants species are able of to grow in contaminated environments by developing several strategies to protect themselves from the chemical toxicity caused by trace metal elements (TME). Recently, some new Cd hyperaccumulators such as *Impatiens glandulifera* (Coakley et al., 2019) and *Eichhornia crassipes* (Eid et al., 2019) have been used for phytoremediation in Cd contaminated soils. Besides, Cd is not an essential element for plant growth (Guo et al., 2019), it is rapidly assimilated by roots and transported to shoots, causing chlorosis, photosynthesis inhibition, biomass reduction (Bankaji et al., 2014), and plant mortality (Zhang et al., 2013).

Endogenous and exogenous levels of plant hormones regulate various mechanisms in plants that help to alleviate Cd‐induced stress in plants (Bali et al., 2019). The involvement of plant phytohormones in the response to trace metal elements in different plant species has been studied. López‐Climent et al. (2011) reported an increase in abscisic acid (ABA) and salicylic acid (SA) contents in citrus plants exposed to high concentrations of Cd^2+^ in the irrigation solution. Cadmium treatment increased endogenous ABA levels in rice plants (Kim et al., 2014). Contrastingly, no significant variation of ABA content in leaves of *Suaeda fruticosa* plants treated with TME elements was found (Bankaji et al., 2014). The ethylene implication in the regulation of signal transduction events during Cd‐induced programmed cell mortality has also been determined and linked to Cd^2+^ tolerance in cultured tomato cells (Iakimova et al., 2008). Despite the efforts to elucidate the phytohormones role in plant responses to TME stress, the mechanisms involved in the interaction of these signaling compounds with the intoxication responses have not been well established. Moreover, contradictory data are often reported. Some studies indicate that auxins can mitigate the trace elements toxic effects in some plants such as sunflower (Fassler et al., 2010). Other reports indicate that in response to Cd^2+^ induced stress, the indole acetic acid (IAA) content decreased significantly in model and cultivated species (Hu et al., 2013). It has been also reported that short‐term cadmium treatment affects IAA homeostasis in barley root tips (Bücker‐Neto et al., 2017).

Plant antioxidant defense system contains both enzymatic and nonenzymatic antioxidants (Sofy et al., 2020). This enzymatic equipment is responsible for the elimination or reduction of oxidative damage under TME stress keeping the balance between ROS production and destruction (Hassan & Mansoor, 2014). Plants have developed a wide range of defense systems to cope with ROS and reduce Cd toxicity (Guo et al., 2019).

Zucchini (*Cucurbita pepo*) is an economically important culture and is grown all over the world for the food supply (as it is a great source of potassium and beta‐carotene), essential oil, and medicinal products (Ayyildiz et al., 2019). Its importance as an economical and medicinal plant is becoming increasingly apparent for its nutrients and bioactive compound richness including phenols, flavonoids, vitamins, amino acids, carbohydrates, and minerals (especially potassium). In addition, it is characterized by its high protein content (28.31%; Badu et al., 2020). Cucurbita species are very different in chemical composition and nutrient content depending on the growing environment, species, or plant/fruit part (Achilonu et al., 2018).

The main objective of this study was to determine the effect of Cd^2+^ on plant physiology, phytohormone patterns of accumulation, and the involvement of antioxidant enzymatic activities in *C. pepo* plants to better understand the adaptation and tolerance mechanisms of this important species to TME‐induced stress conditions.

## 2 MATERIALS AND METHODS

### 2.1 Plant material and culture

Seeds of *C. pepo* (*Cucurbitaceae*) were soaked in distilled water for 2 hr and allowed to germinate in plastic pots containing a mixture of perlite/gravel (in a 2:1 ratio) as inert substrate to avoid interferences with the stress treatment. After 7 days, seedlings were irrigated three times a week with Hewitt nutritive solution's (Hewitt, ), enriched with iron as complex EDTA‐K‐Fe and micronutrients as mixture of salts: MnCl_2_; CuSO_4,_ 5 H_2_O; ZnSO_4_, 7H_2_O; (NH_4_)_6_Mo_7_O_24_, 4 H_2_O; and H_3_BO_3_. After 4 weeks of pretreatment, plants of *C. pepo* were divided into 4 treatment groups of 10 plants that were stressed for 1 month and irrigated (three times a week) with the nutritive solution supplemented with (a) 0 µM CdSO_4_ (control); (b) 100 µM CdSO_4_; (c) 300 µM CdSO_4_; and (d) 500 µM CdSO_4_. The CdSO_4_ solutions were prepared from a salt of cadmium sulfate hydrate (3CdSO_4_, 8H_2_O, Merck). Plants were grown in a greenhouse of the Faculty of Sciences of Bizerte (Tunisia) with the relative humidity between 65% and 92%, the mean temperature (night–day) of 13–24°C, and the natural photoperiod.

To get the fruits, seeds were germinated in peat and seedlings were transferred to perlite 100% as substrate. The seedlings were treated with various Cd‐concentrations (0, 100, 300, and 500 µM) for 10 days after 8 weeks of seed germination. The obtained fruits were separated according to the treatments of Cd^2+^ for the same analysis.

At harvest, the plants were randomly separated in 2 groups of 5 plants for further analysis. In the first group, the leaves were separated from the roots, washed, frozen in liquid nitrogen, and stored at −80°C until the analysis of proline, hydrogen peroxide (H_2_O_2_), malondialdehyde (MDA), enzymatic activity tests, and phytohormone content determination.

The remaining plants were successively rinsed three times with cold water and mopped with filter paper. To eliminate Cd^2+^ adsorbed on the surface of the root, these organs were presoaked in a cold solution of CaCl_2_ for 5 min. The fresh weight and shoot/root biomass ratio were immediately determined.

### 2.2 Tolerance index (TI)

According to Sleimi and Abdely (2003), the tolerance index (in %) was calculated for the entire plant on the basis of fresh weight, using the following formula:

TI=(Biomassoftreatedplants‐biomassofcontrolplant)×100

### 2.3 Trace element determination

300 mg of fresh plant material was digested in Teflon bombs using 3 ml of acid mixture composed with HNO_3_:H_2_SO_4_:HClO_4_ (10:1:0.5; v/v/v) at 110°C for 2 hr (Sghaier et al., 2019). After that, the samples were taken into 50 ml of nitric acid solution at 0.5%. Finally, the extracts were filtered, and concentrations of Cd^2+^ in plant tissues were determined by atomic absorption spectrometry (Perkin Elmer PinAAcle 900T). The blanks, used to set the zero atomic absorption spectrometer, were similarly processed as described above.

### 2.4 Hormone analysis

Phytohormone extraction and analysis were carried out essentially as described in Durgbanshi et al. (2005) with slight modifications (Arbona & Gomez‐Cadenas, 2008). Each plant was processed as a biological replicate, and three independent extractions per plant were performed in each treatment. Briefly, 0.2 g of fresh plant material was extracted in 2 ml of distilled water after spiking with 10 µg of d_2_‐IAA, 100 µg of d_6_‐ABA, 100 µg of ^13^C‐SA, and 100 µg of dihydrojasmonic acid.

After centrifugation at 12,500 rpm at 4°C, supernatants were recovered and pH adjusted to 2.8–3.2 with 30% acetic acid. The acidified water extract was partitioned twice against 2 ml of diethyl ether. The organic layer was recovered and evaporated under vacuum in a centrifuge concentrator (Speed Vac, Jouan). The dry residue was then suspended in water: methanol (9:1) solution. The resulting solution was filtered and directly injected into a HPLC system (Waters Alliance 2695, Waters Corp.). Separations were carried out on a C18 column (Kromasil 100, 5 µm particle size, 100*2.1 mm, Scharlab) using a gradient of methanol and water supplemented with 0.01% acetic acid at a flow rate of 300 µl/min. Hormone content was quantified with a Quattro LC triple quadrupole mass spectrometer (Micromass) connected online to the output of the column through an orthogonal Z‐spray electrospray ion source. Analysis of the phytohormones was based on appropriate Multiple Reaction Monitoring (MRM) of ion pairs for labeled and endogenous jasmonic acid (JA), SA, IAA, and ABA using the following mass transitions: SA 137 > 93, [2H4]‐SA 141 > 97, ABA 263 > 153, [2H6]‐ABA 269 > 159, IAA 174 > 130, [2H2]‐IAA 176 > 132, JA 209 > 59, and [2H6]‐JA 21 > 59 (Arbona & Gomez‐Cadenas, 2008; Durgbanshi et al., 2005). All data were acquired and processed using Mass Lynx v 4.1 software 2.4.

### 2.5 Enzyme assays and analysis

Protein extraction was performed using a ball mill (MillMix20, Domel). Briefly, 100 mg of fresh plant material was extracted in 1.8 ml of buffer extraction containing the potassium phosphate (100 mM, pH 7.5), polyvinyl pyrrolidone, and Triton X. After, the homogenate was centrifuged at 14,000 rpm for 10 min at 4°C, and the supernatant was recovered to measuring the protein contents.

Catalase (CAT) was extracted in 50 mM phosphate buffer (pH 6.8). Homogenates were centrifuged at 2,360 rpm for 45 min at 4°C, and the supernatants were collected. CAT (EC 1.11.1.6) was determined using the hydrogen peroxide‐dependent reduction of titanium chloride.

Superoxide dismutase (SOD) was extracted in 50 mM phosphate buffer (pH = 7.8) with 1.33 mM diethyl‐diamino‐penta‐acetic acid. SOD (EC 1.15.1.1) activity was determined following the O_2_.^‐^ induced reduction of nitroblue tetrazolium using the xanthine–xanthine oxidase system. Absorbance was measured at 560 nm.

Enzymatic activity was expressed as arbitrary units per mg protein. Further details on enzyme assays are given in Arbona et al. (2003).

### 2.6 Proline analysis

Fifty mg of ground material, frozen leaves, or roots was extracted in 5 ml of 3% sulfo salicylic acid (Panreac) by sonication for 30 min. After centrifugation at 4,000 rpm for 20 min at 4°C, extracts were processed as described in Bates et al. (1973) with slight modifications. Briefly, 1 ml of the supernatant was mixed with 1 ml of glacial acetic acid and ninhydrin reagent in a 1:1 (v:v) ratio. The reaction mixture was incubated in a water bath at 100°C for 1 hr. After centrifugation at 2,000 rpm for 5 min at 4°C, absorbance was read at 520 nm. A standard curve was performed with standard proline (Sigma‐Aldrich).

### 2.7 Malondialdehyde analysis

Malondialdehyde content was measured following the procedure of Hodges et al. (1999): 0.2 g of frozen plant material was homogenized in 2 ml of 80% cold ethanol (Panreac) using a tissue homogenizer (Ultra‐Turrax; IKA‐Werke). Homogenates were centrifuged at 4,500 rpm for 20 min at 4°C to pellet debris, and different aliquots of the supernatant were mixed either with 20% trichloroacetic acid (Panreac) or with a mixture of 20% trichloroacetic acid and 0.5% thiobarbituric acid (Sigma‐Aldrich). Both mixtures were incubated in a water bath at 90°C for 1 hr.

Afterward, the samples were centrifuged. The absorbance of the supernatant was read at 440, 534, and 600 nm against a blank. The MDA concentration in the extracts was calculated according to Arbona and Gomez‐Cadenas (2008).

### 2.8 Hydrogen peroxide analysis

Tissue H_2_O_2_ content was estimated according to Brennan and Frenkel (1977). Five hundred mg of frozen leaf or root tissue was macerated in 10 ml cold acetone, and the homogenate was filtered. 2 ml of this filtrate was treated with 1 ml of titanium reagent (20% TiCl_4_ in concentrated HCl, v/v) and 1 ml of concentrated ammonia solution to precipitate the titanium‐hydroper‐oxide complex. After centrifugation (5,000 rpm for 30 min), the precipitate was dissolved in 2N H_2_SO_4_ and the absorbance was read at 415 nm. Hydrogen peroxide content was calculated from a standard curve prepared by using different concentrations of H_2_O_2_ solutions (110–3,520 nmol/ml working solutions prepared from an 888 mM stock solution).

### 2.9 Statistical analysis

All the samples were analyzed at least in three replicates, and the mean values along with standard deviation were shown in bars in the figures. One‐way analysis of variance (ANOVA) was used to determine the statistical significance of the difference between treatments means. ANOVAs were calculated using the Statistica 8. Tukey's HSD test (*p* < .05) was performed to define which specific mean pairs were significantly different.

## 3 RESULTS

### 3.1 Plant morphology and growth

*Cucurbita pepo* demonstrated tolerance to cadmium stress, since all the plants survived, even those threated with the highest Cd^2+^concentration (500 µM). Furthermore, *C. pepo* plants did not show any visible TME toxicity symptoms such as chlorosis and necrosis at 500 µM.

The results show that growth of *C. pepo* plants was significantly affected in treatment with the highest Cd^2+^ concentration (500 µM), exhibiting a significant decrease of 62% in fresh weight (FW) of shoots (Table 1). Likewise, a significant decrease in FW of the roots was observed. This reduction reached 51% at 500 μM of Cd^2+^ compared to the control. The Cd^2+^ treatment affected the shoots/roots ratio in *C. pepo* as it decreased with increasing Cd^2+^ concentration (Table 1).

**TABLE 1:** Biomass production, in shoots (S), roots (R), shoot/root ratio (S/R), and tolerance index (TI) of *Cucurbita pepo* plants under different Cd^2+^ stress treatments

Treatments (Cd, µM)	FW (g)		S/R	TI (%)
	Shoots	Roots		
**0**	30.9 ± 1.7^a^	4.4 ± 0.2^a^	15.1 ± 1.8^a^	100.0 ± 0.0^a^
**100**	30.8 ± 2.0^a^	4.3 ± 0.2^a^	11.9 ± 1.3^a^	103.4 ± 9.9^a^
**300**	18.2 ± 1.3^b^	3.4 ± 0.1^b^	7.1 ± 0.8^b^	62.9 ± 4.9^b^
**500**	11.8 ± 0.7^c^	2.1 ± 0.1^c^	6.0 ± 0.4^b^	40.2 ± 3.2^c^

Note

Different letters denote significant differences at *p* < .05.

In addition, the tolerance index (TI) of *C. pepo* was reduced in the plants grown on stressed media containing higher cadmium concentrations. TI ranged from nearly 100% in 100 μM Cd^2+^ to 40% in 500 μM Cd^2+^ (Table 1).

### 3.2 Cadmium accumulation

**FIGURE 1:**
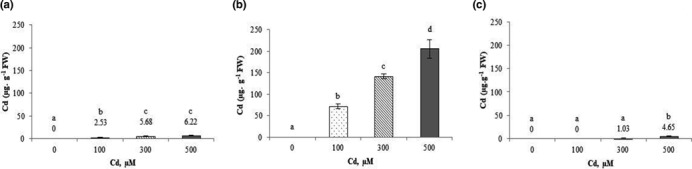
Variation of Cd contents in shoots (a), roots (b), and fruits (c) of *Cucurbita pepo* exposed to different concentrations of Cd^2+^ (0, 100, 300, and 500 µM). Data are mean values of 3 independent determinations ± *SE*. Different letters represent statistical differences at *p* ≤ .05

*Cucurbita pepo*accumulated higher concentrations of Cd^2+^ in the roots than in shoots and fruits. The Cd^2+^ content in the shoots varied from 0 to 6 μg/g FW (Figure 1a), while Cd^2+^ levels in the roots of plants under metallic stress reached 205 μg/g FW, at 500 μM Cd^2+^ (Figure 1b). In fruits, Cd^2+^ content was very low and only detected at concentrations of 300 and 500 µM reaching values of 1.03 and 4.65 µg/g FW, respectively (Figure 1c).

### 3.3 Hormonal content

#### 3.3.1 Salicylic acid

**FIGURE 2:**
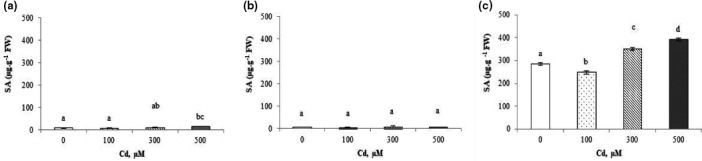
Endogenous levels of salicylic acid (SA) in shoots (a), roots (b), and fruits (c) of *Cucurbita pepo* under Cd^2+^ stress (0, 100, 300, and 500 µM). Data are mean values of 3 independent determinations ± *SE*. Different letters represent statistical differences at *p* ≤ .05

A significant increase in SA concentration in shoots of Cd^2+^ treated plants was observed (Figure 2). In fact, SA content was 2 times higher in 500 µM Cd treated plants (14.89 µg/g of FW) than in controls (7.04 µg/g of FW; Figure 2a). No significant variation in SA concentration was recorded in the roots (Figure 2b).

In fruits, SA concentrations increased significantly with the increasing of the Cd^2+^ irrigation solution concentration. In fact, an increase of 37.7% in the concentration of SA at 500 µM (285.3 µg/g of FW) compared to the control of (392.8 µg/g of FW) was observed (Figure 2c).

#### 3.3.2 Jasmonic acid

**FIGURE 3:**
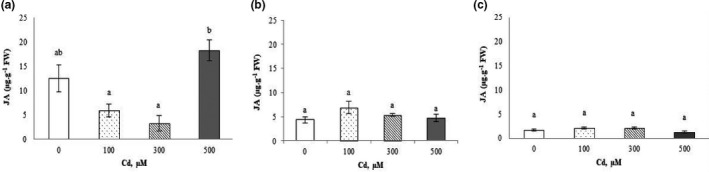
Endogenous levels of jasmonic acid (JA) in shoots (a), roots (b), and fruits (c) of *Cucurbita pepo* under Cd^2+^ stress (0, 100, 300, and 500 µM). Data are mean values of 3 independent determinations ± *SE*. Different letters represent statistical differences at *p* ≤ .05

A significant increase of 45% in the concentration of JA was registered in shoots (Figure 3a; JA varied from 12.56 µg/g of FW in the control to 18.27 µg/g of FW at 500 µM Cd^2+^). On the other hand, the results showed no significant variation of JA levels in roots and in fruits of *C.pepo* treated with Cd^2+^ at different concentrations (Figure 3b,c).

#### 3.3.3 Indole acetic acid

**FIGURE 4:**
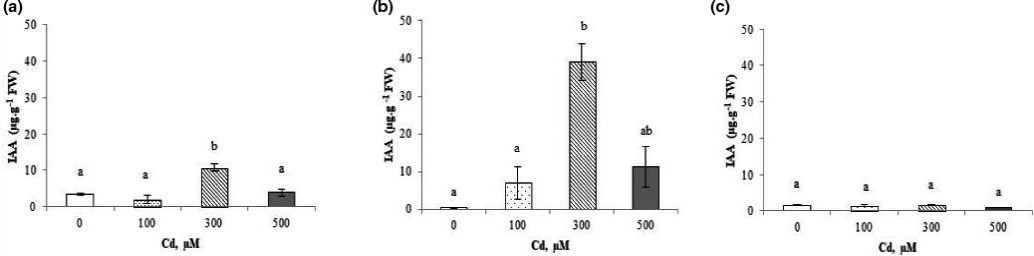
Endogenous levels of indole acetic acid (IAA) in shoots (a), roots (b), and fruits (c) of *Cucurbita pepo* under Cd^2+^ stress (0, 100, 300, and 500 µM). Data are mean values of 3 independent determinations ± *SE*. Different letters represent statistical differences at *p* ≤ .05

Cadmium‐induced stress significantly increased the concentration of IAA in the shoots and roots (Figure 4a,b). The highest concentration was noticed at a concentration of 300 µM Cd^2+^, which attained 10.74 µg/g of FW and 39.15 µg/g of FW in shoots and roots, respectively, compared to the control (3.45 µg/g of FW, 0.49 µg/g of FW), while in fruit, the concentrations of IAA remained in values similar to controls (Figure 4c).

#### 3.3.4 Abscisic acid

**FIGURE 5:**
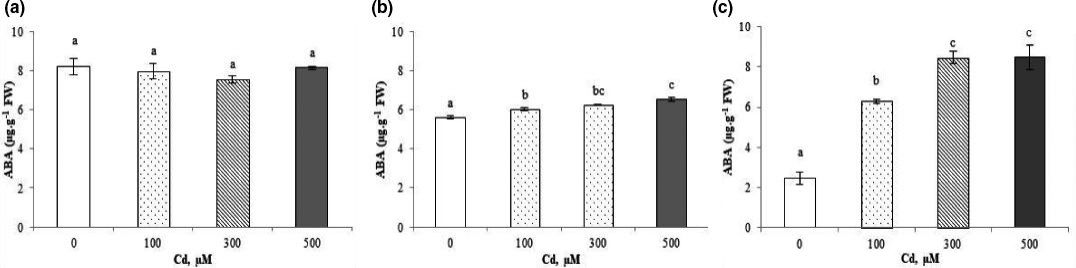
Endogenous levels of abscisic acid (ABA) in shoots (a), roots (b), and fruits (c) of *Cucurbita pepo* under Cd^2+^ stress (0, 100, 300, and 500 µM). Data are mean values of 3 independent determinations ± *SE*. Different letters represent statistical differences at *p* ≤ .05

No significant variation in ABA content in the shoots was detected during the treatments with Cd^2+^ (Figure 5a). Contrariwise, ABA content inroots increased when Cd^2+^ was added to the irrigation solution (it varied from 5.60 µg/g of FW in the control to 6.55 µg/g of FW at 500 µM Cd^2+^; Figure 5b). In fruits, a significant increase in ABA concentration from 2.45 µg/g of FW in the control to 8.47 µg/g of FW at 500 µM of Cd^2+^ was observed (Figure 5c).

### 3.4 Enzymatic activities

**FIGURE 6:**
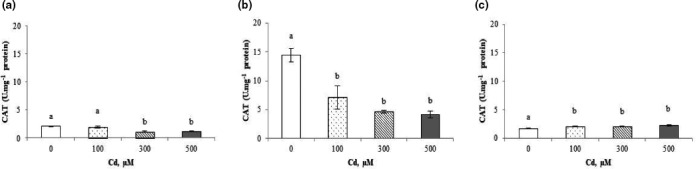
Catalase (CAT) activity in shoots (a), roots (b), and fruits (c) of *Cucurbita pepo* exposed to different concentrations of Cd^2+^ (0, 100, 300, and 500 µM). Data are mean values of 3 independent determinations ± *SE*. Different letters represent statistical differences at *p* ≤ .05

There were variations in the enzymatic antioxidant system activity when plants were exposed to different Cd concentrations. Our results showed that the addition of Cd^2+^ to the irrigation solution induced a significant decrease in CAT activity, both in shoots and roots of *C. pepo* (Figure 6). The reduction was more marked in the roots than in the leaves reaching a decrease of 71.15% and 44.47%, respectively (Figure 6a,b).

A significant increase in the CAT activity as consequence of Cd^2+^ treatment occurred in fruits, which is completely different from the results found in the shoots and the roots (Figure 6c). In fact, CAT activity decreased two times in Cd^2+^ treatment at 300 and 500 µM (Figure 6a,b).

**FIGURE 7:**
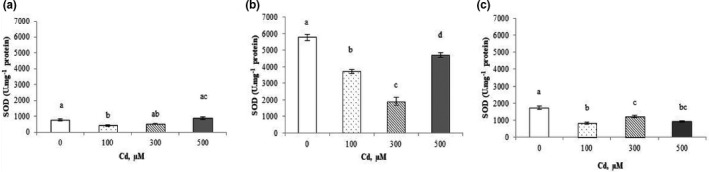
Superoxide dismutase (SOD) activity in shoots (a), roots (b), and fruits (c) of *Cucurbita pepo* exposed to different concentrations of Cd^2+^ (0, 100, 300, and 500 µM). Data are mean values of 3 independent determinations ± *SE*. Different letters represent statistical differences at *p* ≤ .05

The activity of SOD, responsible for the dismutation of superoxide radicals in cells, showed no significant variation in shoots at 500 µM of Cd^2+^ compared to the control (Figure 7a), but decreased significantly, reaching 18% and 46% at 500 µM Cd^2+^, respectively, in roots and fruits (Figure 7b,c).

### 3.5 Malondialdehyde content

The results showed that the MDA content significantly increased in shoots, roots, and fruits (Table 2). The MDA values in the shoots reached 12.13 nmol/g of FW treated with 100 µM of Cd^2+^ compared to the control (10.17 nmol/g of FW), and the rate of increase was approximately 14.71%. MDA content was 18.10 nmol/g of FW in the roots treated with 300 µM of Cd^2+^ compared to the control (10.54 nmol/g of FW), and the rate of increase was approximately 71.75% (Table 2). It should be noted that the production of MDA was higher in fruits, the MDA content increased from 39 nmol/g of FW in the control to 76.53 nmol/g of FW in 500 μM of Cd^2+^.

**TABLE 2:** MDA, Proline, and H_2_O_2_ content in shoots (a), roots (b), and fruits (c) of *Cucurbita pepo* under Cd^2+^ stress

Treatment (µM)	Malondialdehyde nmol/g FW			Proline µmol/g FW			Hydrogen peroxide nmol/g FW		
	Shoots	Roots	Fruits	Shoots	Roots	Fruits	Shoots	Roots	Fruits
**0**	10.17 ± 1.03ab	10.54 ± 0.46^a^	39.0 ± 1.62^a^	0.78 ± 0.06^ab^	0.50 ± 0.02^a^	1.19 ± 0.07^a^	3,502 ± 236.8^a^	804.9 ± 153.9^a^	73.5 ± 7.6^a^
**100**	12.13 ± 0.66^b^	17.49 ± 1.,84^b^	42.5 ± 0.88^a^	0.57 ± 0.10^a^	0.52 ± 0.06^a^	1.86 ± 0.09^bc^	4,025 ± 97.1^a^	1854.8 ± 188.0^b^	137.9 ± 4.0^b^
**300**	11.67 ± 0.63^b^	18.10 ± 1.35^b^	41.9 ± 1.66^a^	0.98 ± 0.03^b^	0.53 ± 0.03^a^	2.15 ± 0.06^c^	2,279 ± 267.5^b^	1,477.8 ± 37.7^ab^	121.0 ± 5.6^b^
**500**	6.72 ± 0.84^a^	12.89 ± 1.67^a^	96.5 ± 2.03^b^	1.50 ± 0.12^c^	0.68 ± 0.11^a^	1.73 ± 0.05^b^	1962 ± 333.9^b^	1,168.5 ± 15.8^ab^	115.5 ± 1.2^b^

Note

Data are mean values of 3 independent determinations ± *SE*. Different letters represent statistical differences at *p* ≤ .05.

### 3.6 Proline

As shown in Table 2, proline content increased significantly in shoots of *C. pepo* with an increase of 93.5% in 500 µM Cd^2+^ treatment (1.49 µmol/g of FW) compared to the control (0.77 µmol/g of FW). Similarly, in fruit, a significant increase in proline content was recorded at a concentration of 300 µM of Cd^2+^ (2.14 µmol/g of FW) compared to the control plant (1.18 µmol/g of FW), and the rate of increase was approximately 81%. In contrast, at the root level, proline content did not show a significant variation.

### 3.7 Hydrogen peroxide

The H_2_O_2_ content decreased significantly in shoots consequence of Cd^2+^ treatment, with a reduction of 43.95% in 500 μM of Cd^2+^ treatment (Table 2). On the contrary, H_2_O_2_ concentration significantly increased in roots compared to the control, reaching the highest content at 100 µM of Cd^2+^, being 45.17% higher than control. Similarly, in fruits and at a concentration of 100 µM of Cd^2+^, there was a significant increase in the H_2_O_2_ content which reaches 137.8 nmol/g of FW compared to the control (73.5 nmol/g of FW).

## 4 DISCUSSION

*Cucurbita pepo* accumulated significant amounts of Cd^2+^ in shoots, roots, and fruits when plants were exposed to the Cd‐containing solution, reaching higher levels in roots than in shoots and fruits (Figure 1). These values are in concordance with those reported by Bankaji et al. (2016) in *Atriplex halimus* after Cd^2+^ exposition. Our results show that most of the absorbed Cd^2+^ is accumulated in the roots of zucchini plants being this is in agreement with other studies, in particular those obtained by Yang et al. (2009). The cadmium accumulation significantly modifies plant growth and development, usually marked by symptoms of toxicity (Dal Corso et al., 2008). These effects could be correlated with an alteration in the absorption and distribution of mineral elements essential for plant function (Dal Corso et al., 2008). This accumulation considerably differs among organs and tissues of the same plant. There is usually less Cd in the leaves than in the roots, and even less in the fruits and seeds (Wagner, 1993).

The determination of fresh weight in the shoots and roots of *C. pepo* shows that the biomass significantly decreased when the concentration of cadmium in the irrigation solution increased (Table. 1). These results are similar to previous studies indicating that cadmium negatively affects plant growth (Shanying et al., 2017), and its toxicity may be the result of perturbation of the balance of some plant hormones (Hasenstein et al., 1988) as well as that the homeostasis of mineral elements (Das et al., 1997).

Few works have been published on the protective impact of SA against TME intoxication (Li et al., 2019; Pàl et al., 2006). The present study shows that Cd^2+^ induced an increase in SA content (Figure 2) in the shoots, roots, and fruits of *C. pepo*, exhibiting a significant correlation between Cd^2+^ and SA content in shoots (*r* = 0.8835) and fruits (*r* = 0.9145; Figure S1a,b). An increase of SA content in leaves of citrus plants grown under Cd^2 +^ stress conditions was observed by López‐Climent et al. (2011). Other authors indicate that, although SA treatment reduces Cd^2+^ uptake by roots, the compound itself has a stressful effect on plants. Thus, the treatment with SA could aggravate the damaging effect of Cd^2+^ in maize (Pàl et al., 2006). These previous reports suggest that SA has a role in the tolerance of plants to trace element stress.

Conflicting results have been observed regarding the response of JA to face Cd stress. Maksymiec et al. (2005) and López‐Climent et al. (2011) demonstrated that Cd^2+^ causes the accumulation of jasmonates in the leaves of citrus, *Arabidopsis thaliana* and *Phaseolus coccineus*. In the present work, a highly significant correlation between Cd^2+^ and JA content in shoots (*r* = 0.9514) was observed (Figure S1c). Similarly, Bankaji et al. (2014) observed increases in the concentration of JA in *Atriplex Halimus* when irrigation solution was supplemented with different Cd^2+^ concentrations.

The IAA content (Figure 4) increased in shoots and roots of *C. pepo* under Cd^2+^ stress. Additionally, a significant correlation between Cd^2+^ and IAA contents in fruits (*r* = 0.9187) has been also observed (Figure S1d). That may suggest an engagement of the IAA in the physiological responses in roots, probably correlated to the new root induction and growth, capable of dealing with stress (Dat et al., 2004).

Abscisic acid content (Figure 5) in roots and fruits of *C. pepo* increased when Cd^2+^ was added to the irrigation solution. In roots, a highly significant correlation between Cd^2+^ and ABA contents (*r* = 0.9934) was observed (Figure S1e), as it has also been previously reported for other plant species (López‐Climent et al., 2011).

To lead oxidative damage, plants activate enzymatic and nonenzymatic antioxidant defense systems that are involved in regulating ROS concentrations (Jalmi & Sinha, 2015; Rizwan et al., 2017).

Superoxide dismutase acts as the first defense line against ROS, dismuting $O_{2}^{‐}$ to H_2_O_2_. However, the product of SOD activity is still toxic (H_2_O_2_) and should be eliminated in subsequent reactions, through the action of CAT and other peroxidases. The equilibrium of SOD and CAT activities is essential in order to determine the steady‐state level of $O_{2}^{‐}$ and H_2_O_2_ (Aschner & Jiang, 2009). The enzymatic system shows variable activity when plants are exposed to different Cd^2+^ concentrations (Pereira et al., 2002). In our study, the SOD activity (Figure 7) increased in shoots, known to be responsible for the dismutation of superoxide radicals from the cells, and decreased in roots and fruits after the increasing of Cd concentrations in the irrigation solutions. Similar results have been revealed by Sandalio et al. (2001) showing that on one hand the treatments with Cd decreased SOD activity in peas, and on the other hand, increased it in mustard (Mobin & Khan, 2007).

The catalase activity recorded in *C. pepo* shoots before Cd treatment can be explained by the existence of the enzyme in the peroxysome to eliminate hydrogen peroxide formed during the photorespiratory cycle as a glycolate oxidase action result (Del Río et al., 2006). The decrease in CAT activity (Figure 6) in shoots and roots after treatment with low concentrations of Cd observed in our study can be explained either by inhibition of the enzyme by the trace metal element or by the elimination of ROS in the roots, as it has been previously reported (Moussa, 2005). Similar, Bankaji et al. (2019) show that the exposure to Zn^2+^‐stress induced a significant decrease in CAT activity in leaves of *Atriplex halimus*. Also, a positive correlation was found between Cd^2+^‐content and CAT activity in shoots (*r* = 0.9616) and roots (*r* = 0.9162). This may reflect an ability of these plants to detoxify H_2_O_2_ even when Cd is present at high concentrations in the irrigation solution (Figure S2a,b). These results are consistent to those of Li et al. (2013) which show that the activity of CAT in the leaves of two plants species tested is diminished in all Cd treatments compared to control.

Cadmium, as other heavy metals, causes the generation of H_2_O_2_ either directly or indirectly through SOD activity. The POD activation is the result of the elimination of H_2_O_2_ removal as a defense reaction (Zhang et al., 2007). When plants are subjected to environmental stresses, the ROS production overcomes the capacity of the antioxidant system. Consequently, oxidative stress occurs resulting in cytotoxic protein and DNA damages as well as lipids peroxidation (Yazici et al., 2007).

Malondialdehyde is one of the end products of lipid peroxidation by free radicals. In this study, MDA content (Table. 2). significantly increased in shoots, roots, and fruits of *C. pepo* in response to cadmium stress. Similarly, Shah et al. (2020) reported higher MDA levels in *Brassica oleracea*, cultivated in the presence of Cd^2+^. Additionally, a highly significant correlation (*r* = 0.9784) between Cd^2+^ and MDA contents in fruits has been observed (Figure S2c).

The accumulation of proline, a compatible osmolyte, under stress conditions is correlated with stress tolerance in a large number of plant species (Kavas et al., 2013). It has also been shown that concentrations are generally higher in stress‐tolerant than in stress‐sensitive plants (Ashraf & Foolad, 2007). Sleimi et al. (2015) indicate that proline plays a role as organic osmoticum in the roots of *Plantago maritima* more so than in its shoots. In our study, *C. pepo* showed a significant increase in proline content (Table. 2) with increasing Cd^2+^doses in the irrigation solution. Our results also show a significant correlation (*r* = 0.8381) between Cd^2+^ and proline contents in the roots of stressed *C. pepo* (Figure S2d). The results of Siddique et al. (2018) showed that increases in proline concentrations help in the elimination of free radicals to make possible osmotic regulation in stressed plants. Oxidative stress‐induced elevations in free proline have been noted in plant species, including sunflower, chickpea, and cucumber (ArikanCeylan et al., 2012; Baloğlu et al., 2012; Sun et al., 2011). Osmoregulation via proline molecules seems as an essential part of the mechanism of protection against metal stress in *C. pepo* plants.

In this work, we report that the production of H_2_O_2_ (Table. 2) significantly increased after different Cd^2+^treatments, regardless trace metal element dose. Therefore, we could conclude that H_2_O_2_ is a signaling molecule, alerting the cell to the presence of an environmental stress (Maksymiec, 2007). According to Dat et al. (2000), H_2_O_2_ can function as a secondary messenger at low concentrations but at high concentrations it becomes toxic. H_2_O_2_ could result from a disproportionate reaction of the superoxide anion by SOD (Mishra et al., 2006). Furthermore, it can also be the result of an electron transport alteration in the photosynthetic and respiratory chains (Gomes‐Junior et al., 2006).

## 5 CONCLUSION

*Cucurbita pepo* has a robust ability to tolerate cadmium stress; as proved by the fact that all plants were able to survive and did not show any visible Cd toxicity symptoms, such as chlorosis, necrosis, or a strong growth inhibition at concentrations up to 300 μM Cd^2+^. Plants accumulated large amounts of Cd^2+^ in roots, suggesting the possibility of having the basic characteristics of a tolerant plant with a high phytostabilization capacity of TME in its underground structures.

However, the high accumulation of Cd^2+^ may be associated with growth inhibition induced by disruption of antioxidant activity. TME tolerance of *C. pepo* plants is, first of all, determined by the barrier function of its root system. This function involves the capacity to accumulate higher concentrations of Cd^2+^ in the roots than in shoots and fruits.

The next level of protection against excess TME involves the phytohormones synthesis. In fact, the strategy of tolerance of *C. pepo* relies IAA accumulation in the roots of plants irrigated with a solution containing high levels of Cd^2+^. This suggests that IAA may be involved in the tolerance of *C. pepo* to trace element stress by stimulating root promotion and growth.

## CONFLICT OF INTERESTS

The authors declare that no conflict of interests exists.

## Supporting information

Figures S1 and S2
