# Supplementary material for: Assessing of growth, antioxidant enzymes, and phytohormone regulation in Cucurbita pepo under cadmium stress
Source: Food Sci Nutr. 2021 Jan 31;9(4):2021–31. doi: 10.1002/fsn3.2169 (PMC8020919; doi:10.1002/fsn3.2169)
Supplement: Supplementary file 1 — Figures S1 and S2 [file FSN3-9-2021-s001.docx]

**Figure S1** The relationship between Cd^2+^ and phytohormones content in *C. pepo* exposed to different concentrations of Cd^2+^ (0, 100, 300 and 500 µM). A: correlation between Cd and SA in shoots, B: correlation between Cd and SA in fruits, C: correlation between Cd and JA in shoots, D: correlation between Cd and IAA in fruits and E: correlation between Cd and ABA in roots.

**Figure S2** The relationship between Cd^2+^ and CAT, Proline and MDA content in *C. pepo* exposed to different concentrations of Cd^2+^ (0, 100, 300 and 500 µM). A: correlation between Cd and CAT in shoots, B: correlation between Cd and CAT in roots, C: correlation between Cd and MDA in roots and D: correlation between Cd and proline in fruits.
